# Supplementary material for: Pattern-enhanced Resonant Soft X-ray Scattering for Operando monitoring of electrochemical solid-liquid interfaces
Source: Nat Commun. 2026 Feb 21;17:2997. doi: 10.1038/s41467-026-69852-9 (PMC13035926; doi:10.1038/s41467-026-69852-9)
Supplement: Supplementary file 1 — Supplementary Information [file 41467_2026_69852_MOESM1_ESM.pdf]

Supplementary Information

Pattern-enhanced Resonant Soft X-ray Scattering for *Operando* Monitoring of Electrochemical Solid-Liquid Interfaces

Haoyi Li[1,8†], Kas Andrle[2†], Qi Zhang[2,3†], Isvar A. Cordova[3,4†], Yao Yang[5], Zhengxing Peng[2], Feipeng Yang[4], Guillaume Freychet[4], Scott Dhuey[6], Alexander Hexemer[4], Brett A. Helms[2,6], Weilun Chao[2,3], Bruno La Fontaine[2,3], Ricardo Ruiz[2,6], Jinghua Guo[4], Wanli Yang[4], Junko Yano[1,7] and Cheng Wang[2,4]*

[1]Liquid Sunlight Alliance, Lawrence Berkeley National Laboratory, Berkeley, California, 94720, United States.

[2]Materials Sciences Division, Lawrence Berkeley National Laboratory, Berkeley, California, 94720, United States.

[3]Center for X-ray Optics, Lawrence Berkeley National Laboratory, Berkeley, California, 94720, United States.

[4]Advanced Light Source, Lawrence Berkeley National Laboratory, Berkeley, California, 94720, United States.

[5]Department of Chemistry and Chemical Biology, Cornell University, Ithaca, New York 14853, United States.

[6]Molecular Foundry, Lawrence Berkeley National Laboratory, Berkeley, California, 94720, United States.

[7]Molecular Biophysics and Integrated Bioimaging Division, Lawrence Berkeley National Laboratory, Berkeley, California, 94720, United States.

[8]Present affiliation: Stanford Synchrotron Radiation Lightsource, SLAC National Accelerator Laboratory, Menlo Park, California, 94025, United States.

*Corresponding author: cwang2@lbl.gov

[†]H.L., K.A., Q.Z. and I.A.C. contributed equally to this work.

Supplementary Table 1. Comparison of Characterization Techniques for Electrochemical Interfaces[1-18].

Technique	PE-RSoXS	TEM	XAS	APXPS	XFM
Dimensional Sensitivity	**Sub-nanometer** (via diffraction-order sensitivity and near-field optical modulation)	**Atomic-scale** (< 0.1 nm) imaging possible	Tens to hundreds of nanometers (limited by beam spot size and detection geometry)	Few nanometers sampling depths in solids; lateral resolution: nanometers to micrometers (limited by beam spot size and detection geometry)	Tens to hundreds of nanometers (microprobe); < 10 nm with ptychography
Temporal Resolution	**Millisecond scale** (single-shot diffraction patterns; compatible with ultrafast detectors)	Millisecond to minutes (with fast cameras)	Seconds to minutes per spectrum (energy scans)	Seconds to minutes; milliseconds possible with fast detectors	Seconds to minutes per scan
Chemical Sensitivity	**High** (tunable to specific absorption edges; diffraction-order selectivity isolates interfacial species)	Limited (requires EELS or EDS)	**High** (direct absorption edge tuning)	**High** (chemical specificity; oxidation states and bonding)	**High** (Element-specific via fluorescence yield)

Interface Accessibility	**High** (coherent amplification of weak buried interfacial signals)	Limited (requires sample thinning or cross-section preparation; interfaces may be altered during the sample preparation)	Moderate (surface-sensitive but buried interfacial signals diluted by the bulk signals)	Moderate (Surface and sub-surface accessible; buried solid−liquid interfaces challenging, limited by probing depth	**High** (Bulk and buried interfaces accessible)
Environment Compatibility	Fully compatible with realistic liquid cells and aqueous conditions	Fully compatible with realistic liquid cells and aqueous conditions	Fully compatible with realistic liquid cells and aqueous conditions	Compatible with near-ambient pressure gases; liquids via microjets or meniscus methods	Compatible with air, vacuum, and liquid cells
Radiation Dose / Damage Risk	**Low** (can be lower than 0.01 $mJ \cdot cm^{-2}$ per frame)	High (beam-induced structural and chemical changes, especially in soft materials)	Moderate dose; soft X-rays more susceptible to chemical changes	Moderate dose; soft X-rays more susceptible to chemical changes	Generally low dose per pixel; scanning can accumulate dose
Statistical measurement	**High** (averages over hundreds of identical nanopattern units depending on the beam spot size)	Limited (small field of view, typically single particles or local regions)	**High** (averages over large illuminated volume)	Moderate (can measure multiple surface regions but limited penetration depth)	**High** (can map statistical areas for a large range)

Measurement Efficiency	**High** (diffraction encodes structural order while resonance tuning provides chemical-state specificity concurrently)	Low (sequential detections with structural and chemical information with EELS/EDS)	Low (primarily provides chemical-state information; structural evolution must be measured separately with other techniques)	Low (primarily provides chemical-state information; structural evolution must be measured separately with other techniques)	Low (primarily elemental mapping, structural information indirect)
Key Strengths	Interface-specific *operando* probing of structural and chemical dynamics; low-dose; diffraction-order selectivity	Direct atomic-scale imaging and crystalline structures	Chemical-state analysis of surfaces and sub-surface regions	Direct surface and sub-surface chemical state under near-realistic conditions	High sensitivity to trace elements; quantitative elemental mapping
Key Limitations	Requires patterned structures for coherent enhancement (patterned templates could be potentially used for supporting powder systems)	Beam-induced changes; limited detection area; difficult to *operando* probe buried interfaces	Limited spatial resolution; bulk-interface separation challenging	Limited penetration depth; challenging for fully buried interfaces	Limited chemical state information; slow scan for high-resolution maps

Supplementary Note 1

Compared to other state-of-the-art interface characterization methods such as transmission electron microscopy (TEM), X-ray absorption spectroscopy (XAS), ambient-pressure X-ray photoelectron spectroscopy (APXPS), and X-ray fluorescence microscopy (XFM), Pattern-Enhanced Soft X-ray Scattering (PE-RSoXS) offers a unique combination of high statistical representativeness, sub-nanometer dimensional sensitivity, and element-specific chemical-state resolution, all achievable under realistic electrochemical operating conditions. Unlike TEM and APXPS, which are limited in probing fully buried solid-liquid interfaces, PE-RSoXS can access these environments without compromising spatial or chemical sensitivity. While XAS provides statistical chemical-state information, it lacks lateral resolution and diffraction-order selectivity for directly probing interfaces, which is inherent to PE-RSoXS through coherent scattering amplification from identical nanopatterns. Furthermore, the ultralow-dose capability of PE-RSoXS ($< 0.01\ mJ \cdot cm^{-2}$ per frame) minimizes radiation-induced artifacts, enabling *operando* monitoring of dynamic processes with millisecond temporal resolution, surpassing the typical time resolution of conventional methods. These combined attributes position PE-RSoXS as a powerful and complementary tool for probing chemical and structural dynamics at buried electrochemical solid-liquid interfaces. PE-RSoXS operates on principles analogous to protein crystallography, where diffraction from periodic units enables reconstruction of real-space features with atomic to nanometer-scale precision. In our case, the periodic nanopatterns define a structural unit cell, and the spatial distribution of chemical species within that unit cell modulates the scattering form factor in a position-sensitive manner. This coherence-based sensitivity to the distribution and geometry of resonant materials enables spatial resolution at sub-nanometer length scales, as supported by both experimental results and the finite-element modeling.

Supplementary Note 2

While diffraction anomalous fine structure (DAFS) has been widely applied in hard X-ray regimes for bulk crystals and epitaxial films[19], its implementation in soft X-rays has been limited due to strong absorption and multiple scattering effects. Compared to traditional DAFS, PE-RSoXS extends this principle by integrating engineered periodic nanopatterns directly into the sample design. Well-ordered line-grating nanopatterns with uniform rectangular geometry were constructed to enhance scattering sensitivity. When the incident soft X-rays are aligned perpendicularly to the grating lines, the periodic structures produce a series of diffraction patterns, as known as "Bragg spots", recorded on a charge-coupled device (CCD) detector.

A unique advantage of PE-RSoXS is the natural alignment of length scales among the soft X-ray wavelength, the engineered nanopatterns, and the interfacial phenomena of interests[20,21]. As a proof of concept, at the Ni L-edge (around 852 eV), the soft X-ray wavelength is around 1.5 nm, which is comparable to the length scale of buried interfacial structures as usually shown in reconstructions and chemical gradients at the solid-liquid boundaries during electroctalysis[22-24]. Meanwhile, the precisely engineered nanopatterns with the width size in order of tens of nanometers, provide a periodic optical modulation that coherently amplifies weak scattering intensity changes of the line-gratings (form factors), especially from the buried electrochemical interfaces[25,26]. This length-scale alignment is a distinctive advantage in optical and infrared methods, which conventionally lack sufficient dimensional sensitivity[27,28]. Besides, hard X-rays have much shorter wavelengths compared to soft X-rays, and they would largely reduce the energy resonance and dimensionally modulated contrast[29,30]. PE-RSoXS bridges this gap and enables *operando* access to structural and chemical dynamics with nanometer to sub-nanometer resolution and element-specific sensitivity at solid−liquid interfaces, respectively.

Supplementary Note 3

We note that RSoXS, and by extension to PE-RSoXS, is fundamentally form-factor sensitive. The scattering intensity arises from the electron density distribution within the scattering object, convoluted with its structural periodicity. The PE-RSoXS technique is not inherently interface-specific, nor does it operate under spectroscopic selection rules like those in nonlinear optics. In our implementation, we deliberately designed Ni line-grating nanopatterns (Ni LGNPs) for PE-RSoXS measurements and they formed a core-shell geometry so that the buried electrochemical solid-liquid interfaces contribute strongly to the form-factor contrast. Perturbations in this shell region under operating conditions, which were confirmed through FEM simulations, selectively influence certain diffraction orders, enabling us to isolate and *operando* monitor interfacial changes. This approach could be equally applied to emphasize bulk or non-interfacial regions, depending on the sample design and modeling strategy.

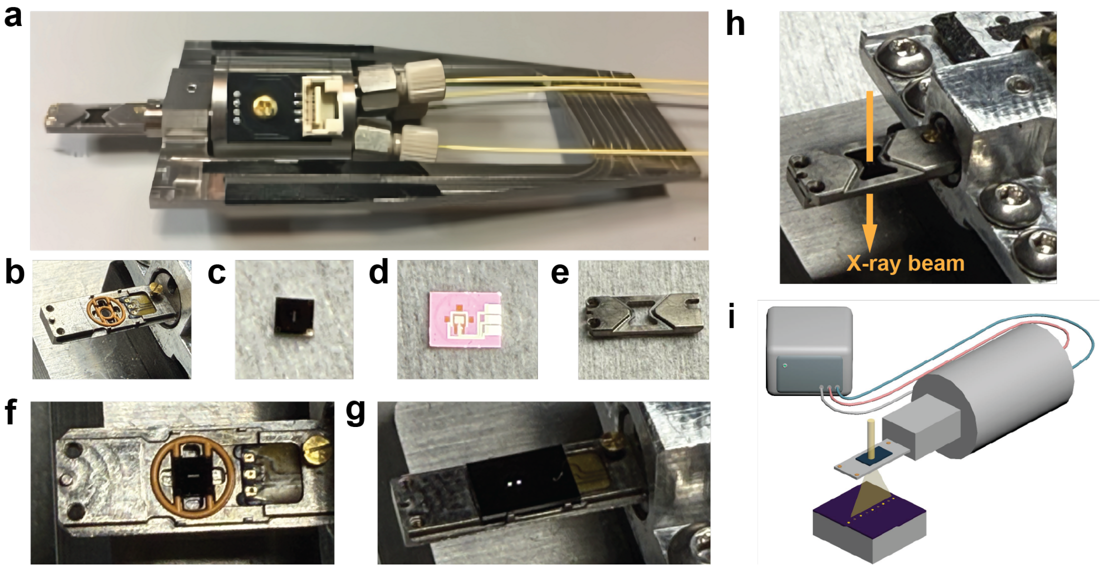

Supplementary Fig. 1. Photos of the customized electrochemical cell for *operando* PE-RSoXS measurements: **a**, the whole cell configuration; **b**, cell inside with an O-ring gasket; **c**, the supporting chip; **d**, the E-chip; **e**, the top sealer; **f**, the supporting chip on top of the O-ring in the cell; **g**, the E-chip on top of the supporting chip in the cell; **h**, the assembled cell with X-ray beam going through. **i**, Schematic illustration of *operando* PE-RSoXS experimental configuration during electrochemical water oxidation.

Supplementary Note 4

The pronounced odd/even diffraction order contrast in this study arises from the deliberate fabrication of Ni line-grating nanopatterns with a 50% duty cycle. In an ideal rectangular profile with sharp edges, this geometry suppresses even orders in the Fourier series representation of the form factor. Subtle structural or compositional perturbations along the line-width direction introduce deviations from ideal structural symmetry in the form factor, leading to a pronounced enhancement of the even-order diffraction signals, while odd orders remain dominated by bulk core contributions[31]. This makes PE-RSoXS highly responsive to structural variations within defined subregions, such as interfacial shells in core-shell architectures. We note that this suppression pattern is specific to the 50% duty cycle case; other fill ratios produce different harmonic effects (e.g., 33% duty cycle structure suppresses every third order), and imperfections such as sidewall angle variation or interfacial roughness can alter the ideal suppression behavior. Our emphasis on odd/even effects here reflects a deliberate design choice to enhance sensitivity to buried interfacial changes, rather than a universal property of Bragg structures.

Supplementary Note 5

In our patterned system, the coherent scattering amplitude from each identical unit adds in phase, resulting in a total amplitude that scales as $N \cdot A$, where N is the number of the identical units. Consequently, the scattered intensity scales as N^2, in contrast to the linear scaling typically observed in systems with randomly distributed or dilute scatterers. This coherent enhancement is distinct from the contrast-driven scattering magnitude ($\Delta\rho^2$ or Δn^2) discussed in small-angle scattering and is central to the concept of PE-RSoXS introduced in this work. In our experiment, with approximately 400 identical Ni line-grating units, it leads to a five-order-of-magnitude enhancement in scattering signal, enabling ultrahigh sensitivity to interfacial changes.

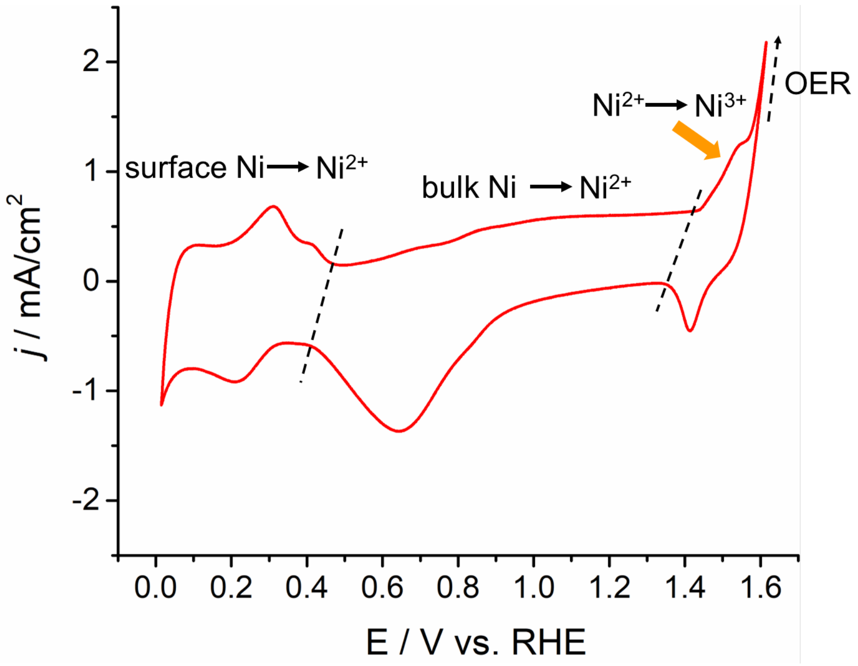

Supplementary Fig. 2. Cyclic voltammetry (CV) profile of Ni metal foam in Ar-saturated 0.1 M NaOH in a standard three-electrode H-cell at 10 mV·s^{-1}. Ni foam, carbon rod and Ag/AgCl in saturated KCl are the working electrode (WE), counter electrode (CE) and reference electrode (RE), respectively. The potentials are calibrated and shown as reversible hydrogen electrode (RHE). The electrochemical oxidation of surface metallic Ni to Ni^{2+} ($Ni(OH)_2$) starts at a potential as low as ~0.1 V and is complete at ~0.4 V. The oxidation of bulk Ni is rather sluggish from ~0.4 to ~1.4 V (vs. RHE) and corresponds to the irreversible reduction peak located at ~0.6 V. The further oxidation of Ni^{2+} to Ni^{3+} (NiOOH) has an onset potential of ~1.4 V. The sharp increase in current density at potential above 1.6 V (vs. RHE) is due to the oxygen evolution reaction (OER) in the alkaline media.

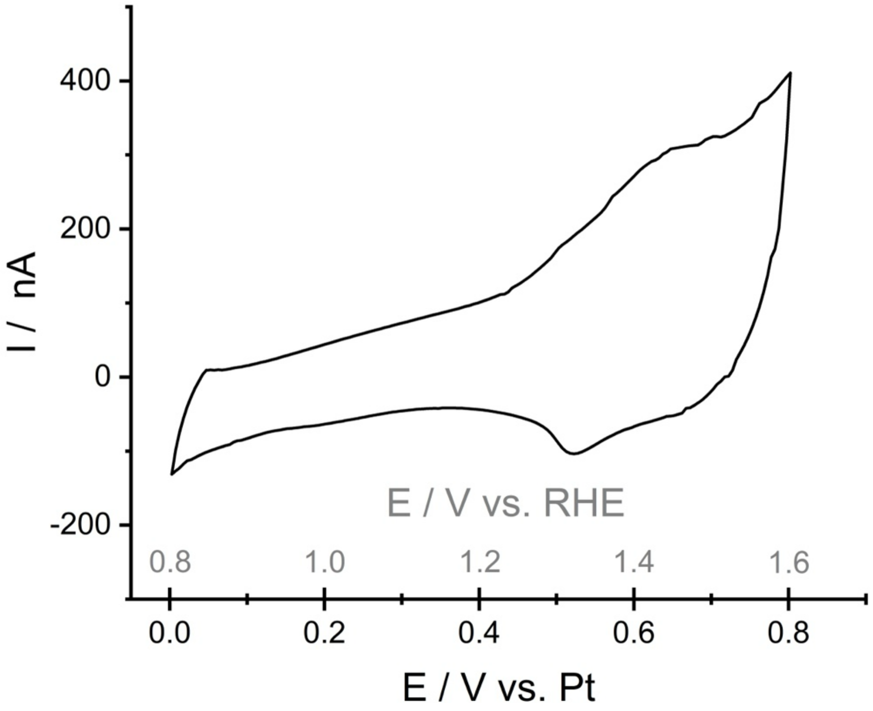

Supplementary Fig. 3. CV profiles of Ni line-grating nanopatterns (LGNPs) deposited on Pt circuits of the E-chip in 0.1 M NaOH collected at 20 $mV{\cdot}s^{-1}$ in the PE-RSoXS electrochemical liquid flow cell. The well-defined CV profile resembles the oxidation and reduction of Ni LGNPs. The potential conversion between Pt and the RHE calibration is 0.8 V based on our previous reports[15,16]. We measured hydrogen adsorption/desorption peaks of the Pt(110) facets at -0.75 V vs. platinum (Pt). Notably, the characteristic peak for Pt(110) typically occurs at approximately 0.1 V vs. RHE. Thus, we estimate that the potential of the Pt pseudo-reference electrode (pseudo-RE) is 0.8 ± 0.1 V.

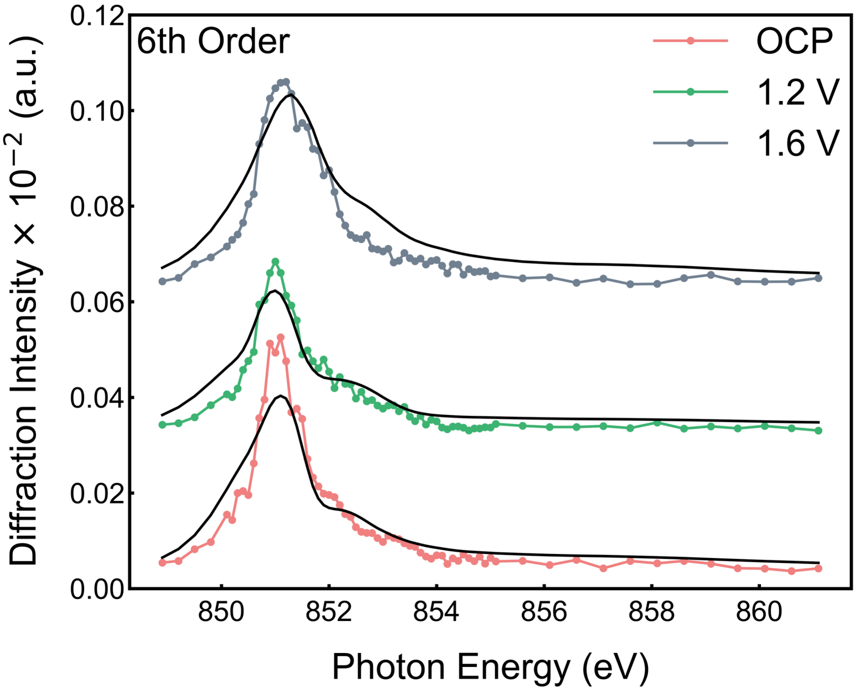

Supplementary Fig. 4. Scattering-derived spectra from the 6th order measured at OCP, 1.2 V and 1.6 V (vs. RHE), respectively, near the Ni L-edge with simulation results based on the models from the FEM shown in **Fig. 2g** of the main text.

Supplementary Note 6

We also considered the possibility of presenting integrated scattering intensity (ISI) curves to reflect the total contrast within the system. However, because our system features a graded and non-binary core-shell geometry with subtle variations in oxidation state, ISI-based methods (which typically integrate $I \cdot \mathbf{q}^2$ across all or bounded $\mathbf{q}$) are not ideally suited for resolving localized compositional changes. Instead, we adopted a FEM-modeling approach, in which the energy-dependent diffraction intensities across multiple orders are simultaneously fit to extract the geometry and optical constants of the shell. This approach enables location-specific reconstruction of $\beta(E)$ (the absorption component), which closely reflects the X-ray absorption behavior of the oxidized Ni species. This framework is consistent with the physical basis reported previously[4,32].

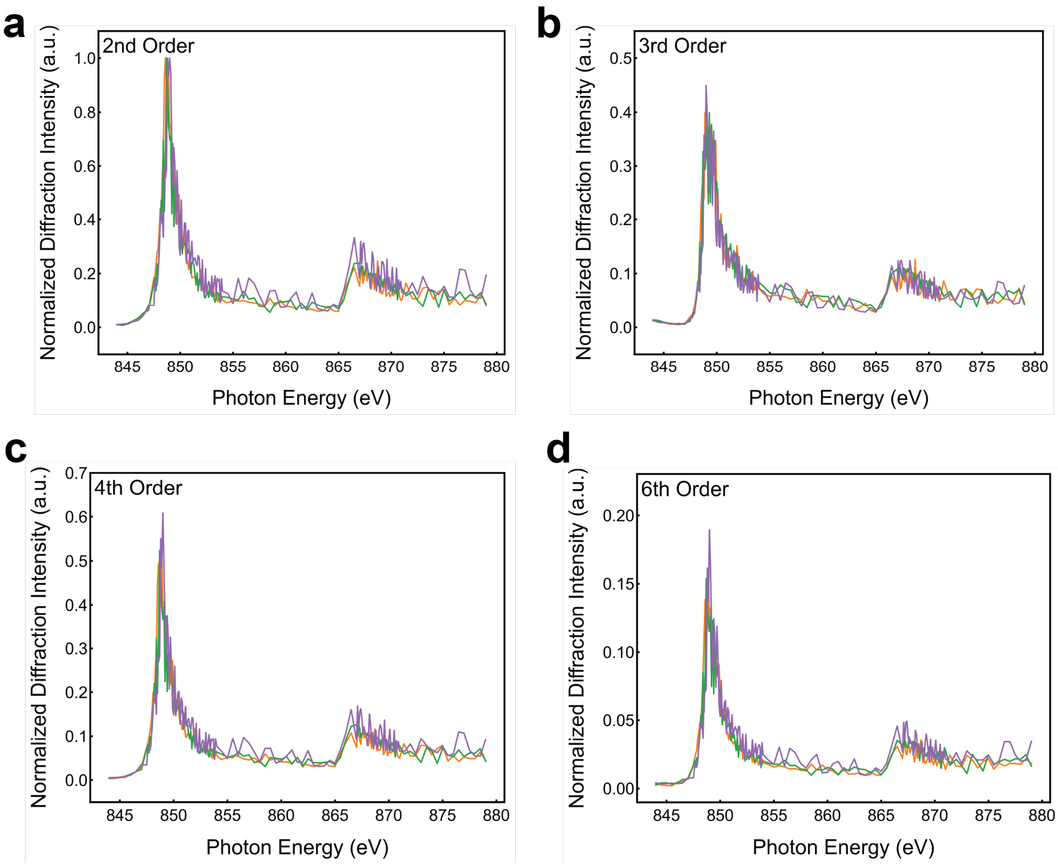

Supplementary Fig. 5. Scattering-derived spectra from the **a**, 2nd, **b**, 3rd, **c**, 4th and **d**, 6th orders acquired on the as-prepared Ni LGNPs. The orange and green curves show consecutive 2 scans in the whole energy range of Ni L-edge with 1 ms of exposure time per frame. The purple curves were obtained from the as-prepared Ni LGNPs after direct beam irradiation for 5 min.

Supplementary Note 7

In this work, the term "non-destructive" refers to the demonstrated ability of PE-RSoXS to acquire high-quality diffraction and spectroscopic data before any measurable X-ray radiation-induced chemical or structural changes occur in the sample. The 1 ms of single-shot exposure time used here was selected as an optimal trade-off between preserving the native state of the solid-liquid interfaces and ensuring sufficient signal-to-noise ratio for reliable data analysis, rather than as the minimum achievable exposure. PE-RSoXS was implemented with such a short single-shot exposure time (1 ms) to enable *operando* monitoring of electrochemical solid-liquid interfaces while minimizing radiation dose (< 0.01 $mJ \cdot cm^{-2}$ per frame) and preserving the native state of the nanopatterns. Although the technique and instrumentation are intrinsically capable of reaching as low as microsecond timescales, the present study focused on a well-characterized Ni LGNPs/OER model system with dynamics in the millisecond regime or slower ones to benchmark dimensional and chemical sensitivity at the buried interfaces under realistic electrocatalytic conditions. This methodological validation provides the foundation for future studies targeting ultrafast processes, such as microsecond electrochemical switching or laser-triggered photoelectrochemical reactions, where the full temporal potential of PE-RSoXS can be exploited.

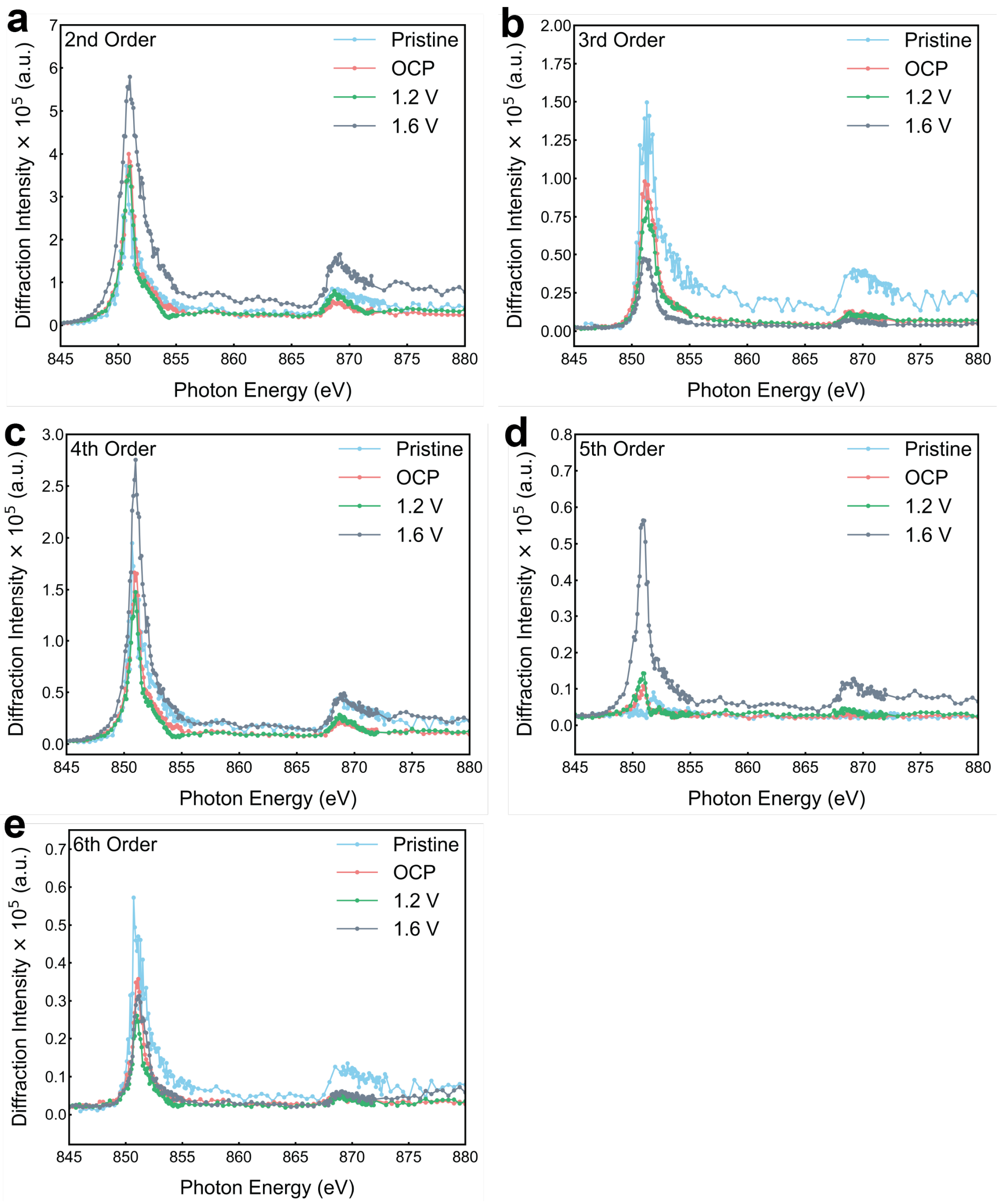

Supplementary Fig. 6. Scattering-derived spectra from the **a**, 2nd, **b**, 3rd, **c**, 4th, **d**, 5th and **e**, 6th orders acquired on the as-prepared Ni LGNPs and Ni LGNPs operated at OCP, 1.2 V, 1.6 V vs. RHE, respectively.

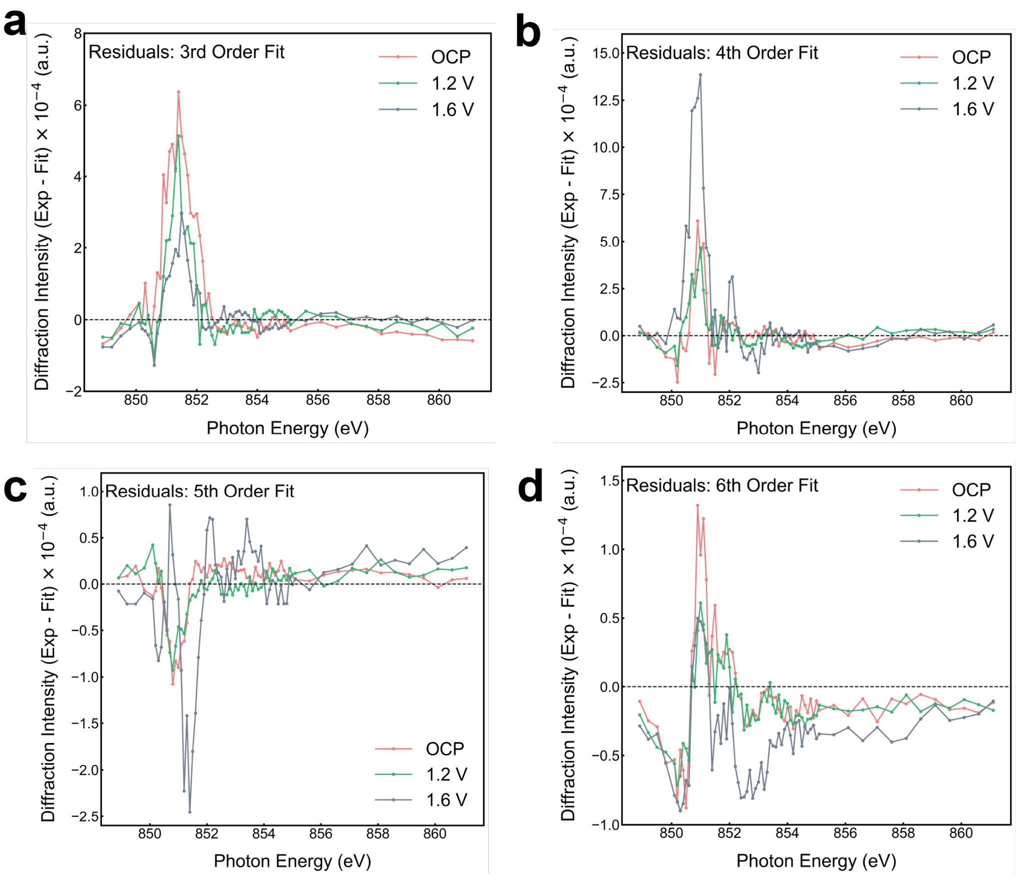

Supplementary Fig. 7. The residual (experimental vs. simulated) spectral intensity as a function of photon energy for the scattering-derived spectra from the a, 3rd, b, 4th, c, 5th and d, 6th orders measured at OCP, 1.2 V and 1.6 V, respectively.

Supplementary Note 8

We note that our simulations assume idealized Ni line-grating structures with smooth interfaces, uniform shell thickness, and perfect sidewall angles. In practice, fabricated and electrochemically evolved nanopatterns may exhibit interfacial roughness, line-edge irregularities, shell thickness inhomogeneity, and sidewall angle variations, etc. Such imperfections can diminish the relative intensity of higher diffraction orders and broaden peaks in **q**-space, effects that are strongly convoluted with shell thickness in the fits. While our current modeling focuses on the dominant parameters of shell thickness and optical constants, these structural deviations could, in principle, lead to misinterpretation of fitted values. We therefore interpret our results within this limitation and note that future extensions to incorporate roughness or inhomogeneity models may help further refine the analysis. This simulation framework is designed to capture the primary spectral trends and diffraction-order dependence, rather than to reproduce every fine spectral detail. Minor discrepancies between simulated and experimental spectra are expected due to interfacial heterogeneity and dynamic restructuring during *operando* conditions, which are not fully incorporated into the current simplified simulation models.

Supplementary Note 9

In this study, the "core width" obtained from resonant scattering fits should be interpreted as an effective domain size rather than a direct physical measurement of the metallic core radius. This effective size is influenced by multiple factors, including changes in material density (especially at 1.2 V and 1.6 V vs. RHE), interface composition, and scattering contrast. Under electro-oxidizing conditions, the simulation models incorporate reduced material density to represent structural hydration, hydroxide incorporation, and increased porosity in the shell region. These density changes alter the core-shell contrast in the scattering profiles, which can lead to slight variations in fitted core width even if the actual metallic core dimension remains unchanged or becomes smaller.

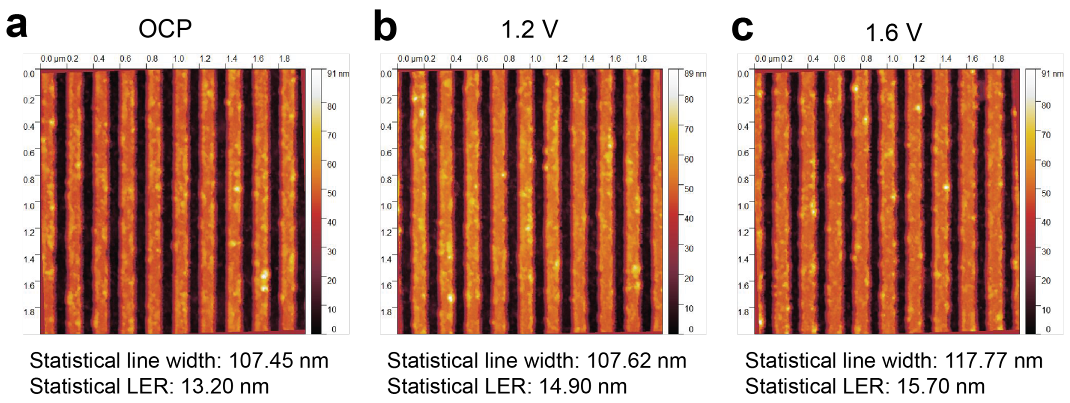

Supplementary Fig. 8. Post-mortem atomic force microscopy (AFM) images of the Ni LGNPs on the SiN_x windows of the sample chip after electrocatalysis at a, OCP, b, 1.2 V and c, 1.6 V, showing the statistical line width and line edge roughness (LER) at each condition.

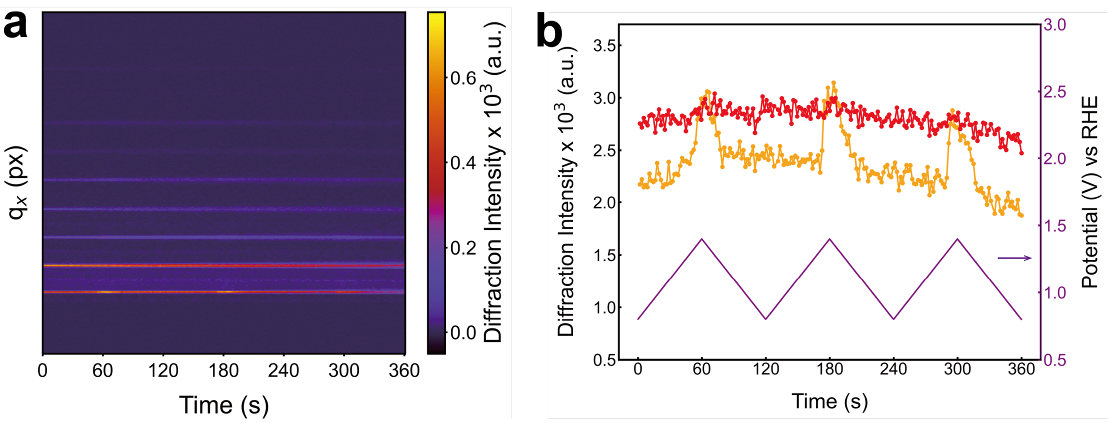

Supplementary Fig. 9. a, Time-dependent diffraction intensity variations on the Ni LGNP electrode over three potential cycling scans between 0.8 V and 1.4 V (vs. RHE) at a scan rate of 10 $mV \cdot s^{-1}$. **b,** Corresponding diffraction intensity changes of the 2nd and 3rd orders in **a** along with the applied potentials.

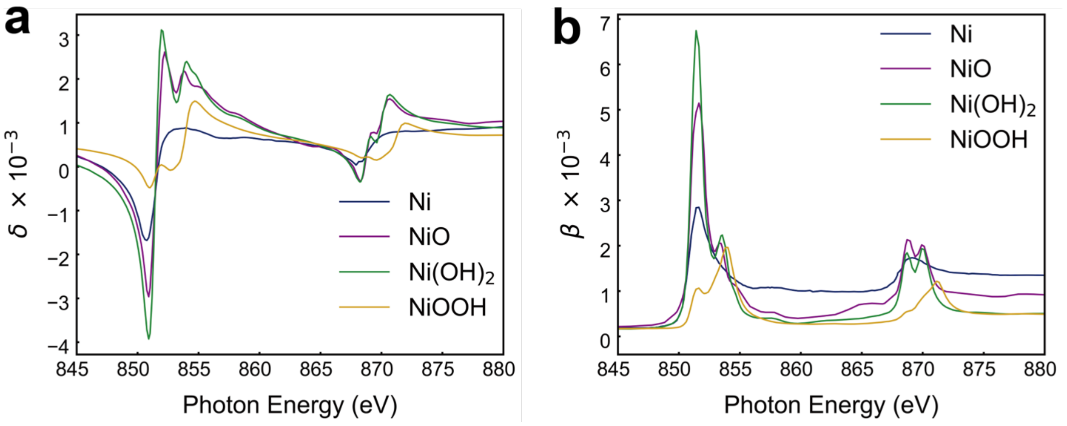

Supplementary Fig. 10. a, Dispersion component, $\delta(E)$ and **b,** absorption component, $\beta(E)$ of the complex refractive index ($n(E) = 1 - \delta(E) + i\beta(E)$) of Ni metal, NiO, $Ni(OH)_2$ and NiOOH standard references as a function of photon energy near the Ni absorption L-edge energy.

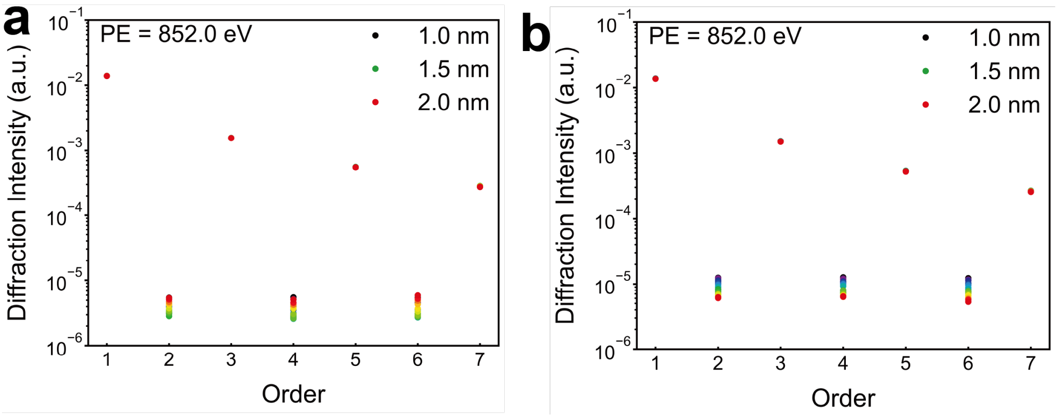

Supplementary Fig. 11. Theoretical FEM-based simulations of diffraction intensities of varied orders based on the model with **a,** a mixture of NiO and NiOOH and **b,** NiOOH in the shell as the shell width increases by 1 nm at 852.0 eV of photon energy.

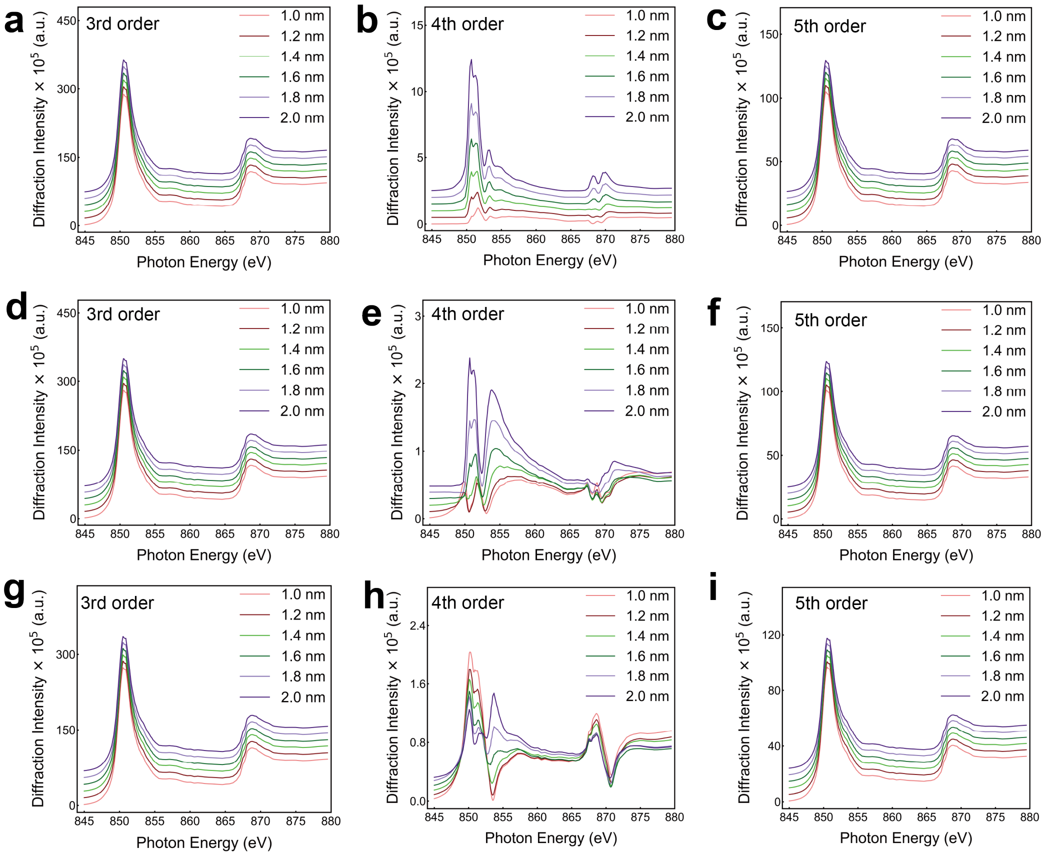

Supplementary Fig. 12. Theoretical FEM-based simulations of spectral variations of the 3rd, 4th, and 5th orders as the shell width increases by 1 nm for the models with the shell composition of **a, b, c,** bare NiO, **d, e, f,** a mixture of NiO and NiOOH, and **g, h, i,** bare NiOOH, respectively.

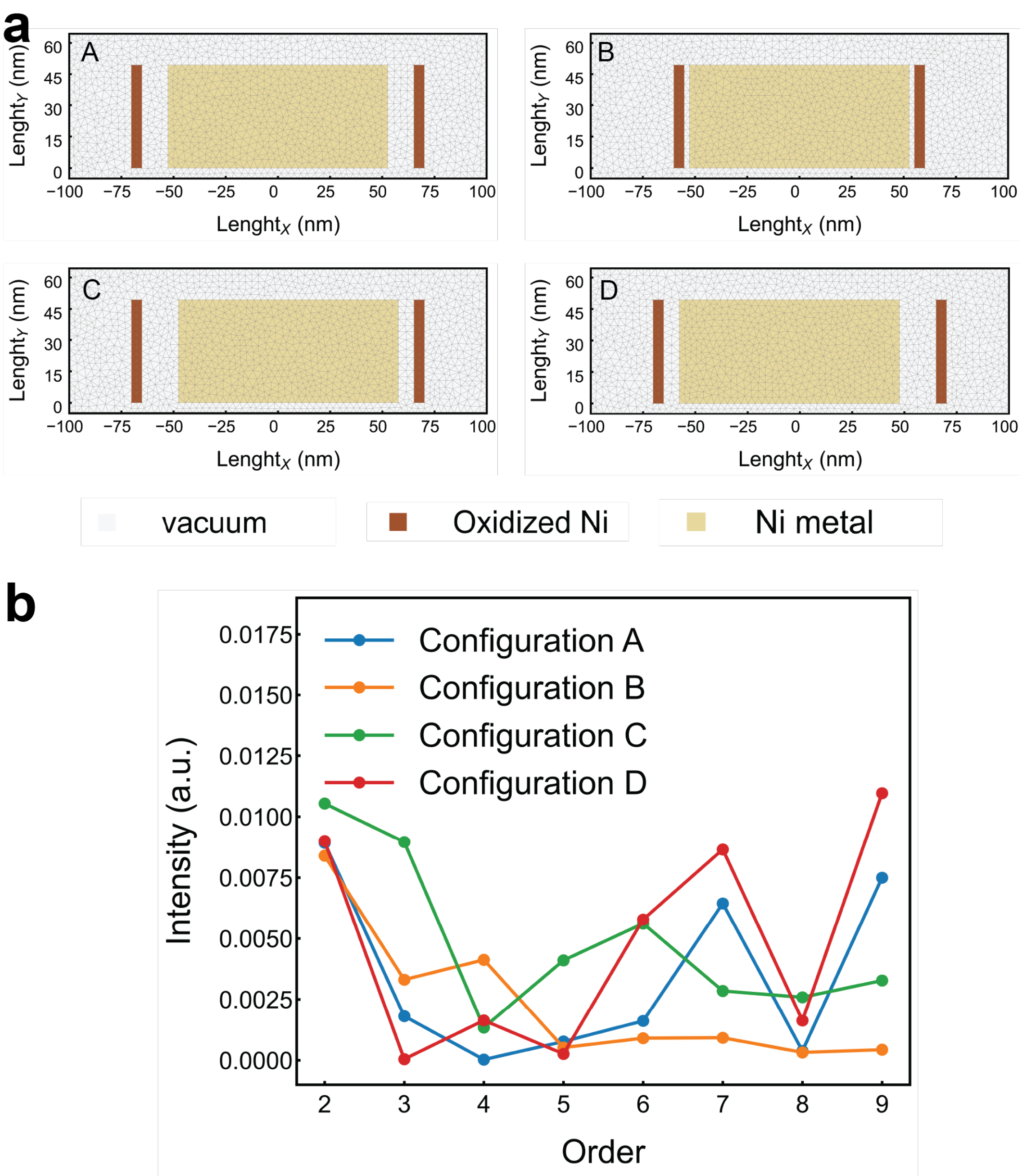

Supplementary Fig. 13. **a**, Four configurations (A to D) of a Ni metal core with oxidized Ni shells at different placements for diffraction intensity simulation. **b**, The simulated diffraction peak intensities of the configurations A to D at 852 eV. The grating cross-section is rectangular, and two smaller rectangular features matching the Ni grating in height, were added to represent the oxide layer. The geometric dimensions of all components were kept constant throughout the study. In configurations A and B, the oxide layers are symmetrically positioned at distances of 15 nm and 5 nm, respectively, from the Ni grating bulk. In configurations C and D, the oxide layers retain the same placement as in configuration A, while the Ni grating is shifted laterally by 5 nm to the right and left, respectively.

References

1. Chen, L. X. *et al.* Deciphering Photoinduced Catalytic Reaction Mechanisms in Natural and Artificial Photosynthetic Systems on Multiple Temporal and Spatial Scales Using X-ray Probes. *Chem. Rev.* **124**, 5421-5469 (2024).
2. Fan, Z. *et al.* In Situ Transmission Electron Microscopy for Energy Materials and Devices. *Adv. Mater.* **31**, 1900608 (2019).
3. Favaro, M. *et al.* Unravelling the electrochemical double layer by direct probing of the solid/liquid interface. *Nat. Commun.* **7**, 12695 (2016).
4. Ferron, T. *et al.* Spectral Analysis for Resonant Soft X-Ray Scattering Enables Measurement of Interfacial Width in 3D Organic Nanostructures. *Phys. Rev. Lett.* **119**, 167801 (2017).
5. Gann, E. *et al.* Soft x-ray scattering facility at the Advanced Light Source with real-time data processing and analysis. *Rev. Sci. Instrum.* **83**, 045110 (2012).
6. Garcia-Esparza, A. T. *et al.* The electrode–electrolyte interface of Cu via modulation excitation X-ray absorption spectroscopy. *Energy Environ. Sci.* **18**, 4643-4650 (2025).
7. Li, H. *et al. Operando* Unveiling of Hydrogen Spillover Mechanisms on Tungsten Oxide Surfaces. *J. Am. Chem. Soc.* **147**, 6472-6479 (2025).
8. Li, H. *et al.* Edge-Exposed Molybdenum Disulfide with N-Doped Carbon Hybridization: A Hierarchical Hollow Electrocatalyst for Carbon Dioxide Reduction. *Adv. Energy Mater.* **9**, 1900072 (2019).
9. Lv, J. *et al.* Low-Dose Electron Microscopy Imaging of Electron Beam-Sensitive Crystalline Materials. *Acc. Mater. Res.* **3**, 552-564 (2022).
10. Magnussen, O. M. *et al.* In Situ and Operando X-ray Scattering Methods in Electrochemistry and Electrocatalysis. *Chem. Rev.* **124**, 629-721 (2024).
11. Pushie, M. J. *et al.* Elemental and Chemically Specific X-ray Fluorescence Imaging of Biological Systems. *Chem. Rev.* **114**, 8499-8541 (2014).
12. Sakdinawat, A. *et al.* Nanoscale X-ray imaging. *Nat. Photonics* **4**, 840-848 (2010).
13. Yang, Y. *et al. Operando* methods: A new era of electrochemistry. *Curr. Opin. Electrochem.* **42**, 101403 (2023).
14. Yang, Y. *et al.* Operando probing dynamic migration of copper carbonyl during electrocatalytic CO_2 reduction. *Nat. Catal.* **8**, 579-594 (2025).
15. Yang, Y. *et al.* Operando studies reveal active Cu nanograins for CO_2 electroreduction. *Nature* **614**, 262-269 (2023).
16. Yang, Y. *et al. Operando* Resonant Soft X-ray Scattering Studies of Chemical Environment and Interparticle Dynamics of Cu Nanocatalysts for CO_2 Electroreduction. *J. Am. Chem. Soc.* **144**, 8927-8931 (2022).
17. Zhang, D. *et al.* Atomic-resolution transmission electron microscopy of electron beam–sensitive crystalline materials. *Science* **359**, 675-679 (2018).
18. Zhang, Q. *et al.* Atomic dynamics of electrified solid–liquid interfaces in liquid-cell TEM. *Nature* **630**, 643-647 (2024).
19. Renevier, H. *et al.* Diffraction anomalous fine-structure spectroscopy at beamline BM2 at the European Synchrotron Radiation Facility. *J. Synchrotron Rad.* **10**, 435-444 (2003).
20. Sunday, D. F. *et al.* Characterizing Patterned Block Copolymer Thin Films with Soft X-rays. *ACS Appl. Mater. Interfaces* **9**, 31325-31334 (2017).

21. Zhong, W. *et al.* Probing morphology and chemistry in complex soft materials with in situ resonant soft X-ray scattering. *J. Phys.-Condens. Mat.* **33**, 313001 (2021).
22. Koelling, S. *et al.* Three-Dimensional Atomic-Scale Tomography of Buried Semiconductor Heterointerfaces. *Adv. Mater. Interfaces* **10**, 2201189 (2023).
23. Yan, P. *et al.* Injection of oxygen vacancies in the bulk lattice of layered cathodes. *Nat. Nanotechnol.* **14**, 602-608 (2019).
24. Yang, A. *et al.* Electrochemical generation of liquid and solid sulfur on two-dimensional layered materials with distinct areal capacities. *Nat. Nanotechnol.* **15**, 231-237 (2020).
25. Barad, H.-N. *et al.* Large Area Patterning of Nanoparticles and Nanostructures: Current Status and Future Prospects. *ACS Nano* **15**, 5861-5875 (2021).
26. Corletto, A. *et al.* Nanoscale Patterning of Carbon Nanotubes: Techniques, Applications, and Future. *Adv. Sci.* **8**, 2001778 (2021).
27. Bai, Y. *et al.* Bond-selective imaging by optically sensing the mid-infrared photothermal effect. *Sci. Adv.* **7**, eabg1559 (2021).
28. Mosca, S. *et al.* Spatially offset Raman spectroscopy. *Nat. Rev. Methods Primers* **1**, 21 (2021).
29. Gaffney, K. J. Capturing photochemical and photophysical transformations in iron complexes with ultrafast X-ray spectroscopy and scattering. *Chem. Sci.* **12**, 8010-8025 (2021).
30. Wang, J. *et al.* In situ X-ray spectroscopies beyond conventional X-ray absorption spectroscopy on deciphering dynamic configuration of electrocatalysts. *Nat. Commun.* **14**, 6576 (2023).
31. Born, M. *et al.* Principles of Optics, 7th (expanded) edition. *United Kingdom: Press Syndicate of the University of Cambridge* **461**, 401-424 (1999).
32. Collins, B. A. *et al.* Resonant soft X-ray scattering in polymer science. *J. Polym. Sci.* **60**, 1199-1243 (2022).
